# p38α plays differential roles in hematopoietic stem cell activity dependent on aging contexts

**DOI:** 10.1016/j.jbc.2021.100563

**Published:** 2021-03-18

**Authors:** Yuriko Sorimachi, Daiki Karigane, Yukako Ootomo, Hiroshi Kobayashi, Takayuki Morikawa, Kinya Otsu, Yoshiaki Kubota, Shinichiro Okamoto, Nobuhito Goda, Keiyo Takubo

**Affiliations:** 1 Department of Stem Cell Biology, Research Institute, National Center for Global Health and Medicine, Tokyo, Japan; 2 Department of Life Sciences and Medical BioScience, Waseda University School of Advanced Science and Engineering, Tokyo, Japan; 3 Division of Hematology, Department of Medicine, Keio University School of Medicine, Tokyo, Japan; 4 School of Cardiovascular Medicine and Sciences, King's College London, London, United Kingdom; 5 Department of Anatomy, Keio University School of Medicine, Tokyo, Japan

**Keywords:** hematopoiesis, hematopoietic stem cells, transplantation, aging, p38MAPK, ataxia–telangiectasia, *Atm*, ataxia–telangiectasia mutated, *Atm*^*fl/fl*^, *Atm*^*flox/flox*^, BM, bone marrow, BMMNCs, bone marrow–derived mononuclear cells, BMT, bone marrow transplantation, 53BP1, tumor suppressor p53-binding protein 1, CAG, chicken β-actin promoter with cytomegalovirus enhancer, GSEA, gene set enrichment analysis, γH2AX, phosphorylated H2A histone family member X, HSC, hematopoietic stem cell, HSPC, hematopoietic stem/progenitor cell, JSPS, Japan Society for the Promotion of Science, LSK, lineage^−^Sca-1^+^c-Kit^+^ cell, LT-HSC, long-term HSC, MEXT, Ministry of Education, Culture, Sports, Science and Technology, MPP, multipotent progenitor cell, *p38α*^*fl/fl*^, *p38α*^*flox/flox*^, PB, peripheral blood, p38MAPK, p38 mitogen-activated kinase, pp38MAPK, phospholyrated-p38MAPK, ROS, reactive oxygen species, TAM, tamoxifen, TNFα, tumor necrosis factor α, qPCR, quantitative PCR

## Abstract

Hematopoietic stem cells (HSCs) and their progeny sustain lifetime hematopoiesis. Aging alters HSC function, number, and composition and increases risk of hematological malignancies, but how these changes occur in HSCs remains unclear. Signaling *via* p38 mitogen-activated kinase (p38MAPK) has been proposed as a candidate mechanism underlying induction of HSC aging. Here, using genetic models of both chronological and premature aging, we describe a multimodal role for p38α, the major p38MAPK isozyme in hematopoiesis, in HSC aging. We report that p38α regulates differentiation bias and sustains transplantation capacity of HSCs in the early phase of chronological aging. However, p38α decreased HSC transplantation capacity in the late progression phase of chronological aging. Furthermore, codeletion of p38α in mice deficient in ataxia–telangiectasia mutated, a model of premature aging, exacerbated aging-related HSC phenotypes seen in ataxia–telangiectasia mutated single-mutant mice. Overall, these studies provide new insight into multiple functions of p38MAPK, which both promotes and suppresses HSC aging context dependently.

Hematopoietic stem cells (HSCs) exhibit self-renewal capacity and multipotency and maintain entire hematopoietic system throughout an organism's life (1). HSCs exist in the G0 phase of the cell cycle at steady state. Upon hematological stress, such as radiation, chemotherapy, and infection, HSCs enter the cell cycle to supply hematopoietic cells to meet organismal hematopoietic demands (2, 3). HSC activation is also seen in bone marrow (BM) transplantation (BMT) as treatment for hematopoietic malignancies and in other diseases. Aging induces functional decline in multiple organ systems, including hematopoiesis. Changes in HSC number and capacity occur as hematopoietic cells age: HSC pool size increases, transplantation capacity decreases, differentiation becomes skewed, and clonality is limited (4, 5, 6, 7, 8). Understanding how hematological stress promotes aging-related changes could suggest treatment for aging-related hematological disease or enhance our ability to expand HSCs *in vitro* without functional decline.

Several stress signals, including p38 mitogen-activated kinase (p38MAPK) signaling, reportedly function in HSC aging. p38MAPK is a member of the MAPK superfamily and includes four isozymes: the major isozyme p38α (*Mapk14*), p38β (*Mapk11*), p38γ (*Mapk12*), and p38δ (*Mapk13*). p38MAPK is activated by DNA damage, reactive oxygen species (ROS), and cytokine release, all induced by stress responses (9, 10). Activated p38MAPK phosphorylates downstream substrates and activates stress responses including cellular senescence (11). In HSCs, BMT or loss of the ataxia–telangiectasia mutated (*Atm*) gene reportedly activates the p38MAPK/p16^Ink4a^/Rb axis *via* ROS induction, decreasing quiescence, and increasing HSC exhaustion (12, 13). Treatment with antioxidants or p38MAPK inhibitors rescues HSC numbers and function, suggesting that p38MAPK compromises HSC function after stress loading (13, 14, 15). However, genetic support for how p38MAPK regulates HSCs *in vivo* is thus far lacking.

Using young p38α knockout mice, we previously reported that p38MAPK activates purine metabolism to promote stress-induced proliferation after transplantation or cytokine stimulation. Therefore, p38MAPK is genetically necessary for HSC proliferation under acute stress conditions in young mice (16). Here, we evaluated effects of p38α loss in models of chronological and premature aging. Relevant to the former, we found that p38α differently modulates HSC function between the early and late progression phases of chronological aging. Moreover, our analysis of effects of p38α loss in *Atm*-deficient mice, which represent a model of premature aging, indicated that p38α sustains pool size and reconstitution capacity in *Atm*-deficient HSCs. Based on these genetic analyses, we conclude that p38MAPK is not simply harmful against aging-related stress but functions as a multimodal factor depending on the context of HSC aging *in vivo*.

## Results

### Loss of *p38α* during the early progression phase of aging does not rescue the aging phenotype in hematopoietic cells

First, we investigated onset of HSC aging in WT mice to select time points appropriate for analysis of HSC aging in *p38α*^*Δ/Δ*^ mice. To do this, we evaluated the number of long-term HSC (LT-HSC) in the BM, along with expression of CD41 and P-selectin (expression of these molecules correlates positively with aging) (17, 18). The number of LT-HSC and expression of CD41 and P-selectin were significantly higher in 1-year-old mice than in 8-week-old mice (Fig. S1, *A*–*C*). Moreover, progression of the HSC aging phenotype (including cell numbers and aging markers) in 2-year-old mice was greater than that in 8-week-old and 1-year-old mice (Fig. S1, *A*–*C*). These results suggest that HSCs acquire the aging phenotype gradually rather than at a certain time; therefore, we selected both 1- and 2-year-old *p38α*^*Δ/Δ*^ mice for analysis of p38MAPK function during the early and late progression phases of HSC aging.

**Figure 1:**
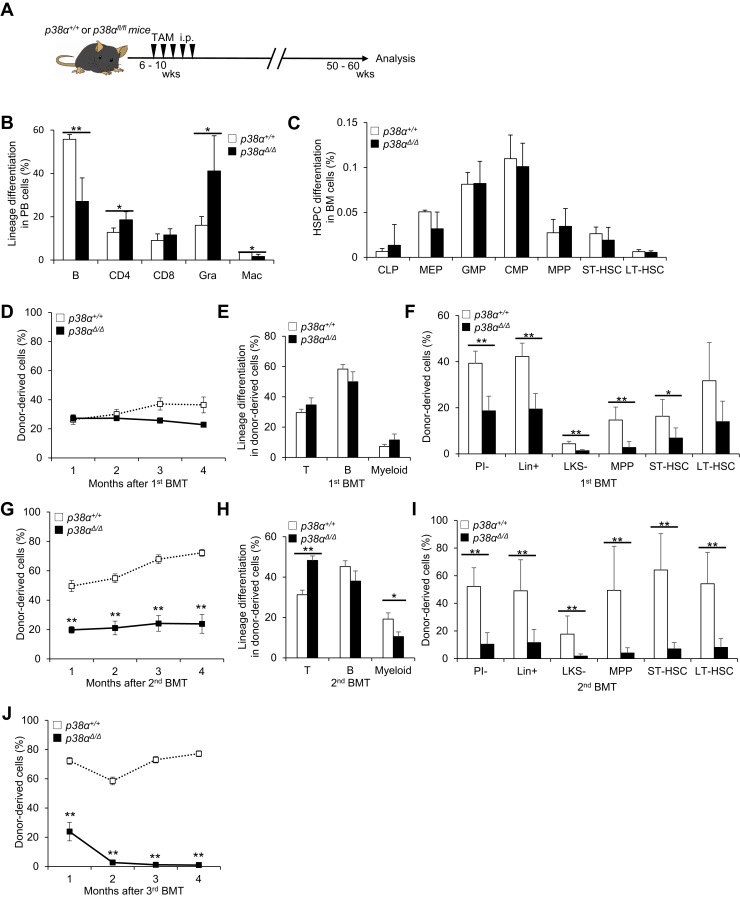
***p38α* loss does not rescue aging phenotypes in hematopoietic cells in 1-year-old mice.***A,* experimental design used to test effects of *p38α* loss in 1-year-old mice. *B* and *C,* analysis of PB differentiation status (B220^+^ B cells [B], CD4^+^ T cells [CD4], CD8^+^ T cells [CD8], Mac-1^+^Gr-1^hi^ granulocytes [Gra] or Mac-1^+^Gr-1^lo^ macrophages [Mac]). *B*, frequencies of BM HSPCs, including CLPs (common lymphoid progenitors; Lin^−^IL7Rα^+^Flt3^+^Sca-1/c-Kit^lo^), MEPs (megakaryocyte-erythroid progenitors; Lin^−^IL7Rα^−^Sca-1^−^c-Kit^+^CD16/32^−^CD34^−^), GMPs (granulocyte-monocyte progenitors; Lin^−^IL7Rα^−^Sca-1^−^c-Kit^+^CD16/32^+^CD34^+^), CMPs (common myeloid progenitors; Lin^−^IL7Rα^−^Sca-1^−^c-Kit^+^CD16/32^−^CD34^+^), MPPs (CD34^+^Flt3^+^LSK), ST-HSCs (CD34^+^Flt3^−^LSK), or LT-HSCs (CD34^−^Flt3^−^LSK) (*C*) in 1-year-old control *p38α*^*+/+*^ or mutant *p38α*^*Δ/Δ*^ mice (means ± SD, n = 5). *D* and *E,* donor-derived chimerism (*D*) and lineage differentiation (CD4/8^+^ T cells [T], B220^+^ B cells [B], or Gr-1/Mac-1^+^ myeloid cells [myeloid]) status (*E*) of PB cells during primary BMT of LT-HSCs from 1-year-old *p38α*^*+/+*^ or *p38α*^*Δ/Δ*^ mice (mean ± SE, n = 4–6). *F,* donor-derived chimerism of BM cells, including LT-HSCs (CD34^−^Flt3^−^LSK), ST-HSCs (CD34^+^Flt3^−^LSK), MPPs (CD34^+^Flt3^+^LSK), LKS^−^ (Lin^−^c-Kit^+^Sca-1^−^), Lin^+^ (lineage marker positive), and PI^−^ (propidium iodide negative) cells from primary recipients at 4 months post-BMT (mean ± SD, n = 5–6). *G* and *H,* donor-derived chimerism (*G*) and lineage differentiation status (*H*) of PB cells after secondary BMT of LT-HSCs from 1-year-old *p38α*^*+/+*^ or *p38α*^*Δ/Δ*^ mice (mean ± SE, n = 4–6). *I,* donor-derived chimerism of BM cells, including LT-HSCs (CD34^−^Flt3^−^LSK), ST-HSCs (CD34^+^Flt3^−^LSK), MPPs (CD34^+^Flt3^+^LSK), LKS^−^ (Lin^−^c-Kit^+^Sca-1^−^), Lin^+^ (lineage marker positive), and PI^−^ (propidium iodide negative) cells, from secondary recipients at 4 months post-BMT (mean ± SD, n = 5–6). *J,* donor-derived chimerism of PB cells after tertiary BMT of LT-HSCs from 1-year-old *p38α*^*+/+*^ or *p38α*^*Δ/Δ*^ mice (mean ± SE, n = 4–6). All data are derived from a single experiment. ∗*p* < 0.05, ∗∗*p* < 0.01. BM, bone marrow; BMT, bone marrow transplantation; HSPC, hematopoietic stem/progenitor cell; LT-HSC, long-term hematopoietic stem cell; MPP, multipotent progenitor cell; PB, peripheral blood; ST-HSC, short-term hematopoietic stem cell; TAM, tamoxifen.

To determine p38MAPK function during aging *in vivo*, we analyzed the effect of conditional loss of *p38α*, the major isozyme in hematopoietic cells (16), in CAG (chicken β-actin promoter with cytomegalovirus enhancer)-CreERT2:*p38α*^*flox/flox*^ (*p38α*^*fl/fl*^) mice. To investigate the early progression phase of aging (from young to 1 year old), we deleted *p38α* (*p38α*^*Δ/Δ*^) from 6- to 10-week-old *p38α*^*fl/fl*^ mice by i.p. injection of tamoxifen (TAM) and then analyzed hematopoiesis at approximately 1 year of age (50–60 weeks) (Fig. 1*A*). First, we analyzed steady-state hematopoiesis and found that *p38α*^*Δ/Δ*^ mice had significantly fewer B cells than controls; however, the percentage of granulocytes in the peripheral blood (PB) was higher than that in controls (Fig. 1*B*). Differentiation status in the BM, spleen, and thymus of *p38α*^*Δ/Δ*^ mice was, however, normal (Figs. 1*C* and S1, *D* and *E*). Next, we tested the functional capacity of *p38α*^*Δ/Δ*^ LT-HSCs by performing serial BMT. Donor-derived PB chimerism in *p38α*^*Δ/Δ*^ cell–transplanted recipients was almost identical to that observed in recipients of *p38α*^*+/+*^ cells (Fig. 1*D*), and the differentiation status of donor-derived PB cells did not differ significantly between the groups (Fig. 1*E*). However, donor-derived BM chimerism was significantly lower in p38α^*Δ/Δ*^ cell–transplanted recipients than in *p38α*^*+/+*^ cell–transplanted recipients (Fig. 1*F*). After secondary transplantation, we observed significantly lower levels of PB chimerism in the *p38α*^*Δ/Δ*^ cell–transplanted group than in the *p38α*^*+/+*^ cell–transplanted group (Fig. 1*G*). Analysis of differentiation status indicated that donor-derived PB cells in *p38α*^*Δ/Δ*^ mice showed lower myeloid chimerism and higher T cell chimerism than those in *p38α*^*+/+*^ cell–transplanted recipients (Fig. 1*H*). Donor-derived BM chimerism was also significantly lower in p38α^*Δ/Δ*^ cell–transplanted recipients than in *p38α*^*+/+*^ cell–transplanted recipients (Fig. 1*I*). Moreover, after tertiary transplantation, *p38α*^*Δ/Δ*^ cells were rarely detected in recipient mice (Fig. 1*J*). These results suggest that p38MAPK activity maintains HSC repopulation capacity and multipotency after transplantation during the early progression phase of chronological aging.

### Deletion of *p38α* improves HSC reconstitution capacity during the late progression phase of aging

**Figure 2:**
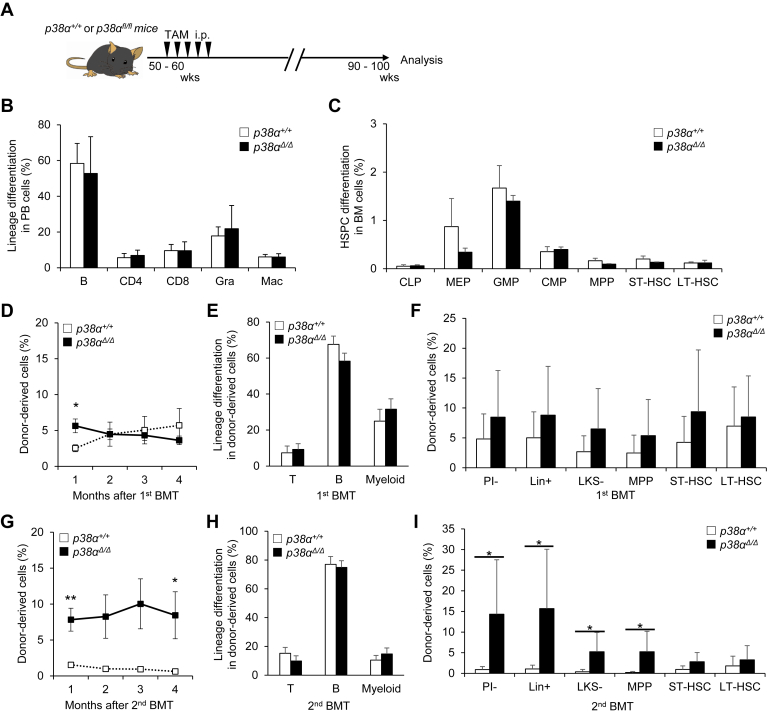
***p38α* loss in the second year of life partially rescues aging phenotypes in HSCs in mice.***A,* experimental design used to test effects of *p38α* loss in the second year of life in mice. *B* and *C,* analysis of PB differentiation status (B220^+^ B cells [B], CD4^+^ T cells [CD4], CD8^+^ T cells [CD8], Mac-1^+^Gr-1^hi^ granulocytes [Gra], or Mac-1^+^Gr-1^lo^ macrophages [Mac]) (means ± SD, n = 7–9) (*B*), and frequencies of BM HSPCs including CLPs (common lymphoid progenitors; Lin^−^IL7Rα^+^Flt3^+^Sca-1/c-Kit^lo^), MEPs (megakaryocyte-erythroid progenitors; Lin^−^IL7Rα^−^Sca-1^−^c-Kit^+^CD16/32^−^CD34^−^), GMPs (granulocyte-monocyte progenitors; Lin^−^IL7Rα^−^Sca-1^−^c-Kit^+^CD16/32^+^CD34^+^), CMPs (common myeloid progenitors; Lin^−^IL7Rα^−^Sca-1^−^c-Kit^+^CD16/32^−^CD34^+^), MPPs (CD34^+^Flt3^+^LSK), ST-HSCs (CD34^+^Flt3^−^LSK), or LT-HSCs (CD34^−^Flt3^−^LSK) (means ± SD, n = 3–5) (*C*) in 2-year-old *p38α*^*+/+*^ or *p38α*^*Δ/Δ*^ mice. *D,* chimerism of donor-derived PB cells in primary BMT of LT-HSCs from 2-year-old *p38α*^*+/+*^ or *p38α*^*Δ/Δ*^ mice (means ± SE, n = 7–8). *E,* lineage differentiation (CD4/8^+^ T cells [T], B220^+^ B cells [B], or Gr-1/Mac-1^+^ myeloid cells [myeloid]) in PB cells from primary recipients 4 months after BMT (means ± SE, n = 7–8). *F,* chimerism of donor-derived BM cells, including LT-HSCs (CD34^−^Flt3^−^LSK), ST-HSCs (CD34^+^Flt3^−^LSK), MPPs (CD34^+^Flt3^+^LSK), LKS^−^ (Lin^−^c-Kit^+^Sca-1^−^), Lin^+^ (lineage marker positive), and PI^−^ (propidium iodide negative) cells, from primary recipients 4 months after BMT (means ± SD, n = 7–8). *G,* chimerism of donor-derived PB cells after secondary BMT of LT-HSCs from 2-year-old *p38α*^*+/+*^ or *p38α*^*Δ/Δ*^ mice (mean ± SE, n = 7–8). *H,* lineage differentiation (CD4/8^+^ T cells [T], B220^+^ B cells [B], or Gr-1/Mac-1^+^ myeloid cells [myeloid]) of PB cells from secondary recipients at 4 months post-BMT (mean ± SE, n = 7–8). *I,* chimerism of donor-derived BM cells, including LT-HSCs (CD34^−^Flt3^−^LSK), ST-HSCs (CD34^+^Flt3^−^LSK), MPPs (CD34^+^Flt3^+^LSK), LKS^−^ (Lin^−^c-Kit^+^Sca-1^−^), Lin^+^ (lineage marker positive), and PI^−^ (propidium iodide negative) cells from secondary recipients at 4 months post-BMT (mean ± SD, n = 7–8). All data are derived from a single experiment. ∗*p* < 0.05 and ∗∗*p* < 0.01. BM, bone marrow; BMT, bone marrow transplantation; HSPC, hematopoietic stem/progenitor cell; LT-HSC, long-term hematopoietic stem cell; MPP, multipotent progenitor cell; ST-HSC, short-term hematopoietic stem cell; TAM, tamoxifen.

To evaluate the phenotypes associated with the late progression phase of chronological aging, we attempted to generate 2-year-old *p38α*^*Δ/Δ*^ mice that had been treated with TAM at around 10 weeks of age. However, this was not possible because the mice did not survive to an age of 2 years (Fig. S2*A*). Therefore, we injected *p38α*^*fl/fl*^ mice aged approximately 1 year (50–60 weeks) with TAM to delete *p38α* and then analyzed those mice and corresponding controls 1 year later (*i.e.*, at 2 years of age) (Fig. 2*A*). Deletion of *p38α* from mice injected with TAM at approximately 1 year of age was confirmed by genomic quantitative PCR (qPCR) performed when the *p38α*^*Δ/Δ*^ and control mice reached 2 years of age. The results showed that *p38α* expression in *p38α*^*Δ/Δ*^ was up to 70% lower than that in *p38α*^*+/+*^ mice (Fig. S2*B*). Evaluation of steady-state hematopoiesis in *p38α*^*Δ/Δ*^ and control *p38α*^*+/+*^ mice indicated comparable differentiation status and hematopoietic stem/progenitor cell (HSPC) numbers in PB, BM, spleen, and thymus (Figs. 2, *B* and *C* and S2, *C*–*I*). We then tested functional capacity of HSCs from mutant and control mice by performing serial BMT. Donor-derived PB and BM chimerism and differentiation status in primary recipients did not differ significantly between genotypes (Figs. 2, *D*–*F* and S2*J*). By contrast, after secondary BMT, *p38α*^*Δ/Δ*^ mice showed comparable differentiation status in PB but higher relative PB chimerism than did the control *p38α*^*+/+*^ cell–transplanted group (Figs. 2, *G* and *H* and S2*K*). Furthermore, *p38α*^*Δ/Δ*^ cell–transplanted groups showed higher BM chimerism of multipotent progenitor cells (MPPs) and a greater degree of differentiation in secondary recipients (Fig. 2*I*), supporting the idea that *p38α* deletion improves overall repopulation capacity after transplantation in the later phase of aging. Expression levels of *p16*^*Ink4a*^ and *p19*^*Arf*^, tumor suppressors that are reportedly activated in stressed HSCs (13), did not differ between *p38α*^*+/+*^ and *p38α*^*Δ/Δ*^ HSCs at steady state or after secondary BMT (Fig. S2, *L* and *M*). These results suggest that p38MAPK activity decreases HSC repopulation capacity after transplantation in the late progression phase of chronological aging.

### Deletion of *p38α* suppresses inflammation and phenotypically revitalizes old HSCs

**Figure 3:**
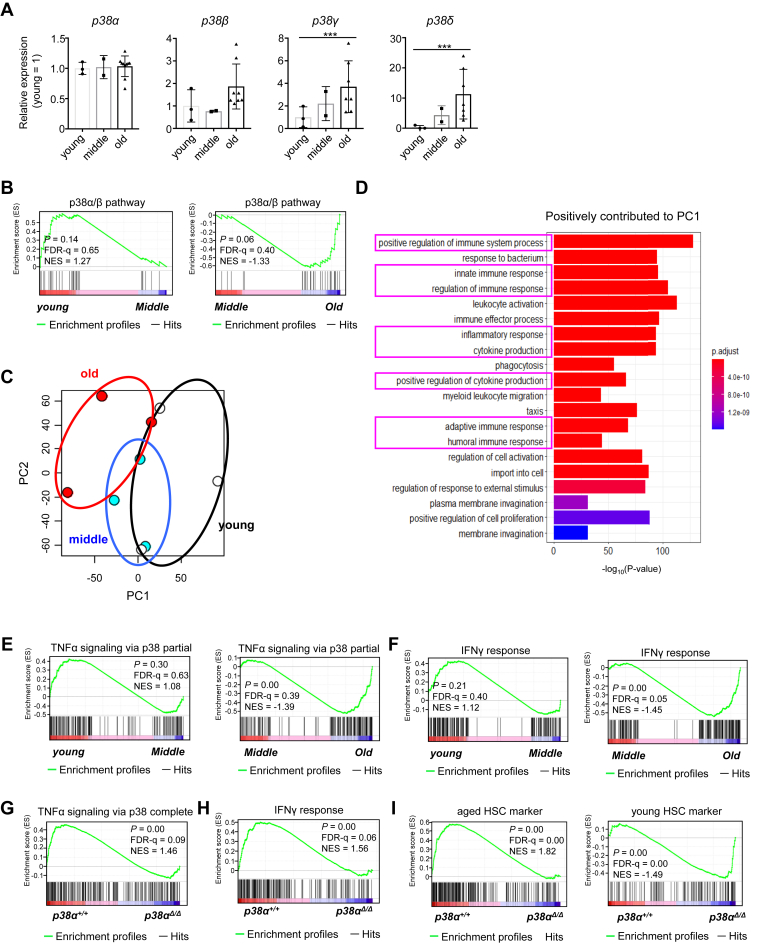
**Loss of *p38α* prevents inflammation of hematopoietic stem cells (HSCs) aged 1 to 2 years old and rejuvenates the phenotype of old HSCs.***A,* RT-PCR analysis of *p38α*, *p38β*, *p38γ*, and *p38δ* expression in young (10-week-old), middle-aged (1-year-old), and old (2-year-old) LT-HSCs (CD48^−^CD150^+^LSK). Data are expressed as fold induction relative to expression in young HSCs (mean ± SD, n = 2–10). *B,* GSEA plots for middle-aged (1-year-old) and old (2-year-old) HSCs showing genes involved in the p38α/β pathway. *C,* principal component (PC) analysis of young (2 months old), middle-aged (12 months old), and old (24 months old) HSCs. *D,* gene ontology analysis of genes involved in biological process that make a positive contribution in PC1. *E,* GSEA plots of young (2 months), middle-aged (12 months), and old (24 months) HSCs showing genes involved in TNFα responses *via* p38 partial. *F,* GSEA plots of young (2 months), middle-aged (12 months), and old (24 months) HSCs showing genes involved in the interferon γ (IFNγ) pathway. *G* and *H,* GSEA plots of *p38α*^*+/+*^ or *p38α*^*Δ/Δ*^ LT-HSCs collected from 2-year-old mice showing genes involved in TNFα responses *via* p38 complete (*G*) and IFNγ pathways (*H*). *I,* GSEA of *p38α*^*+/+*^ or *p38α*^*Δ/Δ*^ LT-HSCs showing genes upregulated in aged HSCs and young HSCs. All data are derived from a single experiment. ∗∗∗*p* < 0.001. FDR, false discovery rate; GSEA, gene set enrichment analysis; LT-HSCs, long-term hematopoietic stem cells; TNFα, tumor necrosis factor α.

Because the effect of *p38α* on HSC regulation changes with age, we compared expression of p38MAPK isozymes in LT-HSCs at different time points during aging. We found that expression of *p38α* did not change with age (Fig. 3*A*). However, we found that expression of *p38γ* (*Mapk12*) and *p38δ* (*Mapk13*) was higher in old HSCs than in young and middle-aged HSCs, even though relative expression levels (*i.e.*, compared with that of the internal control gene) were quite low (Fig. S3*A*). To evaluate changes in p38MAPK gene expression patterns with age, we performed gene set enrichment analysis (GSEA) of young (10 weeks), middle-aged (1 year), and old (2 years) LT-HSCs using transcriptome data downloaded from the Gene Expression Omnibus database (GSE151333). Gene sets related to p38MAPK downstream pathways were significantly enriched in old HSCs compared with middle-aged HSCs; however, there was no difference between young and middle-aged HSCs (Fig. 3*B*). These findings indicate that p38MAPK signaling increases with age, especially between middle and old age.

Next, we investigated potential transcriptional changes related to *p38α* function during aging. Heat map analysis identified age-related differentially expressed genes (Fig. S3, *B* and *C* and Tables S1 and S2). In addition, principal component analysis indicated that HSCs display different transcriptomic landscapes at different time points (Fig. 3*C*). Transcriptional differences identified by principal component analysis were clearly characterized by principal component 1, which mediates inflammatory responses and cytokine production (Fig. 3*D*). GSEA also showed that many gene sets, including those related to the MAPK pathway and inflammatory pathways, are enriched in old HSCs compared with middle-aged HSCs (Fig. S3*D*). Notably, gene sets related to inflammation, including tumor necrosis factor α (TNFα) and interferon responses, were significantly enriched in old to middle-aged HSCs but not in middle-aged and young HSCs (Figs. 3, *E* and *F* and S3*E*). These findings indicate that aging is not a linear event, and that inflammation is activated to a greater extent in the phase from middle to old age than in the phase from young to middle age.

Studies show that p38MAPK triggers production of proinflammatory cytokines, such as TNFα, interleukin-1, and interleukin-6 upon stress stimulation (19, 20). Therefore, we hypothesized that p38MAPK, especially *p38α*, is responsible for activating inflammatory signaling in hematopoietic cells in old mice. To address this hypothesis, we performed transcriptome analysis of LT-HSCs collected from 2-year-old *p38α*^*+/+*^ and *p38α*^*Δ/Δ*^ mice and examined the effects of *p38α* on inflammation. For these analyses, experiments with *p38α*^*Δ/Δ*^ mice were conducted within the time frame shown in Figure 2*A*. We found that *p38α*^*Δ/Δ*^ HSCs showed significantly lower expression of p38MAPK pathway–related genes than *p38α*^*+/+*^ HSCs (Fig. S3*F*). Gene sets related to TNF responses, interferon responses, and other inflammatory pathways were downregulated in *p38α*^*Δ/Δ*^ HSCs compared with *p38α*^*+/+*^ HSCs (Figs. 3, *G* and *H* and S3*G*). In addition, gene expression of aged HSC markers was significantly downregulated, and that of young HSC markers was significantly upregulated, in *p38α*^*Δ/Δ*^ HSCs compared with *p38α*^*+/+*^ HSCs (Fig. 3*I* and Table S3) (21). Taken together, these results suggest that *p38α* deficiency alters expression of gene programs associated with HSC aging and inflammation and results in protection of HSC aging during the late progression phase of chronological aging.

### *p38α* loss does not rescue defective repopulating capacity in *Atm*^*Δ/Δ*^ HSCs

**Figure 4:**
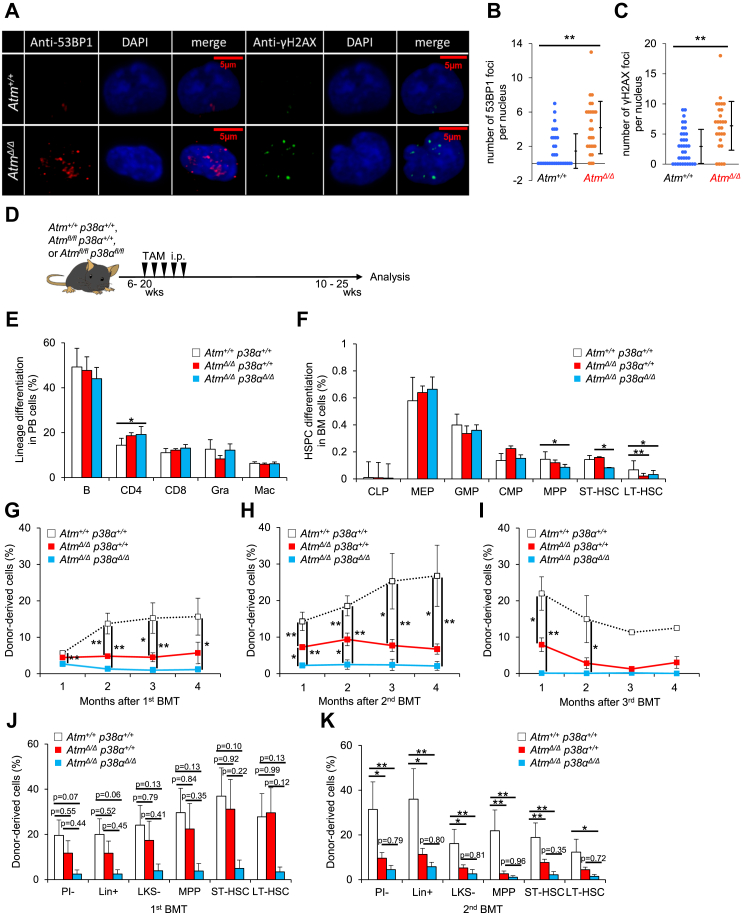
**Codeletion of *Atm* and *p38α* induces HSC loss and defective transplantation capacity.***A*–*C,* immunocytochemical analysis of 53BP1 and gamma-H2AX in *Atm*^*+/+*^ or *Atm*^*Δ/Δ*^ LT-HSCs recovered from BMT recipients. Cytospin samples of sorted cells were stained with anti-53BP1 (*red*) and gamma-H2AX (*green*) antibodies combined with DAPI (*blue*) (*A*). Quantification of 53BP1 (*B*) and gamma-H2AX foci (*C*) (means ± SD, n = 27–35). *D,* experimental design used to test effects of *p38α* loss in *Atm*^*Δ/Δ*^ mice. E and *F,* analysis of PB differentiation status (B220^+^ B cells [B], CD4^+^ T cells [CD4], CD8^+^ T cells [CD8], Mac-1^+^Gr-1^hi^ granulocytes [Gra], or Mac-1^+^Gr-1^lo^ macrophages [Mac]) (means ± SD, n = 5) (*E*), and frequencies of BM HSPCs including CLPs (common lymphoid progenitors; Lin^−^IL7Rα^+^Flt3^+^Sca-1/c-Kit^lo^), MEPs (megakaryocyte-erythroid progenitors; Lin^−^IL7Rα^−^Sca-1^−^c-Kit^+^CD16/32^−^CD34^−^), GMPs (granulocyte-monocyte progenitors; Lin^−^IL7Rα^−^Sca-1^−^c-Kit^+^CD16/32^+^CD34^+^), CMPs (common myeloid progenitors; Lin^−^IL7Rα^−^Sca-1^−^c-Kit^+^CD16/32^−^CD34^+^), MPPs (CD34^+^Flt3^+^LSK), ST-HSCs (CD34^+^Flt3^−^LSK), or LT-HSCs (CD34^−^Flt3^−^LSK) (means ± SD, n = 5) (*F*) in mice of indicated genotypes. *G*–*I,* chimerism of donor-derived PB cells in primary (*G*), secondary (*H*), and tertiary (*I*) BMT of HSCs of indicated genotypes (means ± SE, n = 6). *J* and *K,* chimerism of donor-derived BM cell, including LT-HSCs (CD34^−^Flt3^−^LSK), ST-HSCs (CD34^+^Flt3^−^LSK), MPPs (CD34^+^Flt3^+^LSK), LKS^−^ (Lin^−^c-Kit^+^Sca-1^−^), Lin^+^ (lineage marker positive), and PI^−^ (propidium iodide negative) cells from primary (*J*) and secondary (*K*) recipients, 4 months after BMT (means ± SE, n = 5–6). All data are derived from a single experiment. ∗*p* < 0.05 and ∗∗*p* < 0.01. *Atm*, ataxia–telangiectasia mutated; BM, bone marrow; BMT, bone marrow transplantation; 53BP1, tumor suppressor p53-binding protein 1; DAPI, 4′,6-diamidino-2-phenylindole; HSPC, hematopoietic stem/progenitor cell; LT-HSC, long-term hematopoietic stem cell; MPP, multipotent progenitor cell; PB, peripheral blood; ST-HSC, short-term hematopoietic stem cell; TAM, tamoxifen.

We next employed a different model to investigate how *p38α* loss affects HSCs under pathological conditions such as progeria syndrome. The gene *Atm* is reportedly responsible for ataxia–telangiectasia in humans, and *Atm* knockout mice show some premature aging-like phenotypes, such as decreased survival and increased expression of *p16*^*Ink4a*^ and *p19*^*Arf*^ in some organs (22). To evaluate whether conditional *Atm*-deficient (CAG-CreERT2) HSCs phenocopy the germ line *Atm*-deficient HSCs, we focused on DNA repair, as there are reports that germ line *Atm* loss impairs DNA repair in HSCs (2, 23), and analyzed DNA damage accumulation by counting phosphorylated H2A histone family member X (γH2AX) and tumor suppressor p53-binding protein 1 (53BP1) foci in *Atm*^*Δ/Δ*^ and WT HSCs 1 week after transplantation. The number of respective γH2AX and 53BP1 foci was significantly increased in *Atm*^*Δ/Δ*^ HSCs relative to controls, suggesting that *Atm* loss impairs DNA repair similarly to the germ line *Atm* loss (Fig. 4, *A*–*C*). Here, to assess p38MAPK function in *Atm*-deficient mice, we compared phenotypes seen in *CAG-CreERT2::Atm*^*flox/flox*^ (*Atm*^*fl/fl*^) and *CAG-CreERT2::Atm*^*fl/fl*^*p38α*^*fl/fl*^ mice. To do so, we injected TAM into age-matched 6- to 20-week-old mice of both genotypes to delete floxed genes in respective mice, hereafter referred to as *Atm*^*Δ/Δ*^ and *Atm*^*Δ/Δ*^*p38α*^*Δ/Δ*^ mice, and then analyzed phenotypes at 10 to 25 weeks of age (Fig. 4*D*). TAM-administered Cre(−)::*Atm*^*fl/fl*^ or *Atm*^*fl/fl*^*p38α*^*fl/fl*^ mice (*Atm*^*+/+*^ or *Atm*^*+/+*^*p38α*^*+/+*^) served as controls.

We first focused on steady-state hematopoiesis. Differentiation status in PB and cell number in BM were almost identical in all three genotypes (Figs. 4*E* and S4*A*). However, we observed a significant decrease in LT-HSC subsets both in *Atm*^*Δ/Δ*^ and *Atm*^*Δ/Δ*^*p38α*^*Δ/Δ*^ relative to control mice (Fig. 4*F* and S4*B*). Furthermore, relative to controls, frequencies of MPP subsets and short-term HSCs were significantly reduced in *Atm*^*Δ/Δ*^*p38α*^*Δ/Δ*^ mice and more mildly in *Atm*^*Δ/Δ*^ mice (Fig. 4*F* and S4*B*). Next, we performed serial BMT to assess LT-HSC repopulating ability in these models. Differentiation status was comparable, but donor-derived PB chimerism in *Atm*^*Δ/Δ*^ and *Atm*^*Δ/Δ*^*p38α*^*Δ/Δ*^ cell–transplanted recipients was significantly lower than the control *Atm*^*+/+*^*p38α*^*+/+*^ cell–transplanted group (Figs. 4*G* and S4*C*). Donor-derived BM chimerism of *Atm*^*Δ/Δ*^ and *Atm*^*Δ/Δ*^*p38α*^*Δ/Δ*^ cell–transplanted recipients showed trends similar to those in PB cells and did not differ significantly from primary BMT (Fig. 4*J*). PB trends similar to primary BMT were also seen in secondary and tertiary BMTs (Fig. 4, H and I). Furthermore, we observed lower relative PB and BM chimerism in *Atm*^*Δ/Δ*^*p38α*^*Δ/Δ*^ relative to *Atm*^*Δ/Δ*^ cell–transplanted recipients (Fig. 4*K*). Similarly, donor-derived PB cells showed lower T cell chimerism in *Atm*^*Δ/Δ*^ relative to control *Atm*^*+/+*^*p38α*^*+/+*^ cell recipients and much lower T cell chimerism in *Atm*^*Δ/Δ*^*p38α*^*Δ/Δ*^ relative to *Atm*^*Δ/Δ*^ (Fig. S4*D*). These results show that although *Atm* loss compromises HSC number and function, *p38α* has a protective function in *Atm*^*Δ/Δ*^ mice, resulting in further HSC dysfunction in double knockout.

### Inducible *Atm* loss does not alter activation of the p38MAPK/ROS pathway at steady state but induce p38MAPK activation after transplantation in HSCs

**Figure 5:**
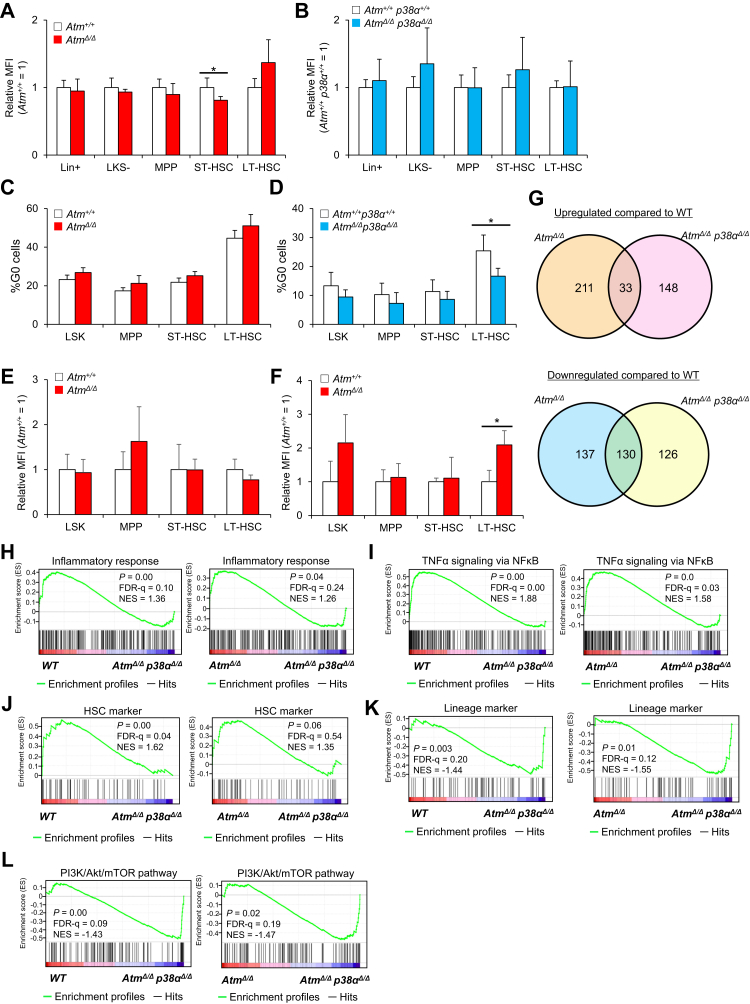
**Inducible loss of *Atm* or *Atm/p38α* does not alter the ROS pathway and cell cycle status.***A* and *B,* redox-sensitive MitoTracker Orange CMH_2_TMROS fluorescence in LT-HSCs (CD34^−^Flt3^−^LSK), ST-HSCs (CD34^+^Flt3^−^LSK), MPPs (CD34^+^Flt3^+^LSK), LKS^−^ (Lin^−^c-Kit^+^Sca-1^−^), Lin^+^ (lineage marker positive) in *Atm*^*+/+*^ or *Atm*^*Δ/Δ*^ mice (*A*) and *Atm*^*+/+*^*p38α*^*+/+*^ or *Atm*^*Δ/Δ*^*p38α*^*Δ/Δ*^ mice (*B*) (mean ± SD, n = 4). Six- to 20-week-old *CAG-CreERT2::Atm*^*flox/flox*^ and *CAG-CreERT2::Atm*^*flox/flox*^*p38α*^*flox/flox*^ were treated with TAM and then analyzed 4 to 5 weeks later. Age-matched TAM-administrated controls (Cre(−)::*Atm*^*flox/flox*^ or *Atm*^*flox/flox*^*p38α*^*flox/flox*^ mice) were served as controls. *C* and *D,* cell cycle status of LT-HSCs (CD34^−^Flt3^−^LSK) in *Atm*^*+/+*^ or *Atm*^*Δ/Δ*^ mice (*C*) and *Atm*^*+/+*^*p38α*^*+/+*^ or *Atm*^*Δ/Δ*^*p38α*^*Δ/Δ*^ mice (*D*). Shown are results of flow cytometric analysis of Ki67 (mean ± SD, n = 4). *E* and *F,* p38MAPK phosphorylation status in LT-HSCs (CD34^−^Flt3^−^LSK), ST-HSCs (CD34^+^Flt3^−^LSK), MPPs (CD34^+^Flt3^+^LSK), LSK (Lin^−^c-Kit^+^Sca-1^+^) in mice of indicated genotypes at steady state (*E*) and 3 days after 4 Gy radiation (*F*). Mean fluorescence intensity (MFI) was analyzed by intracellular flow cytometry (means ± SD, n = 4). *G,* summary of microarray analysis of *Atm*^*Δ/Δ*^ or *Atm*^*Δ/Δ*^*p38*^*Δ/Δ*^ LT-HSCs. Venn diagrams represent the number of genes upregulated (up) or downregulated (down) at least eightfold. *H*–*L,* GSEA plots for gene sets corresponding to the inflammatory response (*H*), HSC markers (*I*), and proliferation and lineage markers (*J*), lineage markers (*K*), and the PI3K/Akt/mTOR pathway (*L*) in *Atm*^*+/+*^ (WT) and *Atm*^*Δ/Δ*^*p38*^*Δ/Δ*^, or *Atm*^*Δ/Δ*^ and *Atm*^*Δ/Δ*^*p38*^*Δ/Δ*^ LT-HSCs (CD34^−^Flt3^−^LSK). All data are derived from a single experiment. ∗*p* < 0.05. *Atm*, ataxia–telangiectasia mutated; FDR, false discovery rate; LT-HSC, long-term hmatopoietic stem cell; MPP, multipotent progenitor cell; mTOR, mechanistic target of rapamycin; ST-HSC, short-term hematopoietic stem cell; TNFα, tumor necrosis factor α.

Previous studies report that activation of p38MAPK in HSCs induces HSC defects in *Atm*^*Δ/Δ*^ mice by increasing production of ROS and subsequently upregulating *p16*^*Ink4a*^ and *p19*^*Arf*^ (13). Therefore, we asked whether ROS production and p16^Ink4a^ and p19^Arf^ activation occurred following *Atm* deletion *in vivo* in both *p38α* WT and mutant backgrounds. Flow cytometric and qPCR analyses showed that ROS production and *p16*^*Ink4a*^ and *p19*^*Arf*^ expression were comparable in *Atm*^*Δ/Δ*^, *Atm*^*Δ/Δ*^*p38α*^*Δ/Δ*^, and control HSCs (Figs. 5, *A* and *B* and S5, *A* and *B*). ROS-p16^Ink4a^/p19^Arf^ signaling reportedly disrupts cell cycle quiescence in HSCs (24). Thus, we analyzed cell cycle status in *Atm*^*Δ/Δ*^ and *Atm*^*Δ/Δ*^*p38α*^*Δ/Δ*^ HSCs using Ki67 staining. However, frequencies of Ki67-negative quiescent (G_0_) cells were unchanged in *Atm*^*Δ/Δ*^ and *Atm*^*+/+*^ HSCs (Fig. 5*C*). By contrast, frequencies of Ki67-negative cells were significantly lower in *Atm*^*Δ/Δ*^*p38α*^*Δ/Δ*^ relative to *Atm*^*+/+*^*p38α*^*+/+*^ cells (Fig. 5*D*). These results indicate that ROS-p16^Ink4^/p19^Arf^ signaling is not activated *in vivo* by *Atm* deletion in HSCs. Next, to determine whether p38MAPK activation is induced after *Atm* deficiency, we measured phospholyrated-p38MAPK (pp38MAPK) levels in *Atm*^*Δ/Δ*^ LT-HSC and non-HSCs at steady state. Phosflow analysis revealed that pp38MAPK levels were unchanged relative to WT controls in *Atm*^*Δ/Δ*^ HSCs and non-HSCs at steady state (Figs. 5*E* and S5*C*). A previous study reported that p38α activation in HSPCs occurs immediately after hematological stress and returns to normal levels in a short period (16). Therefore, we evaluated pp38MAPK levels in *Atm*^*Δ/Δ*^ and *Atm*^*+/+*^ HSPCs at 3 and 7 days after BMT using phosflow. pp38MAPK levels were significantly higher in *Atm*^*Δ/Δ*^ LT-HSC at day 3, but no significant difference was observed between two genotypes at 7 days following transplantation (Figs. 5*F* and S5, *C* and *D*). These results suggested that, after conditional deletion of *Atm*, activation of p38MAPK or ROS-p16^Ink4^/p19^Arf^ signaling is unchanged in steady state, whereas activation of p38MAPK is increased in *Atm-*deficient HSCs after transplantation.

### Deletion of *p38α* in *Atm*-deficient HSCs impairs stemness through inflammatory pathway

Finally, to determine how p38α functions in *Atm*^*Δ/Δ*^ HSCs, we performed transcriptome analysis of HSCs collected from 11-week control *Atm*^*+/+*^ (WT), *Atm*^*Δ/Δ*^, and *Atm*^*Δ/Δ*^*p38α*^*Δ/Δ*^ mice to identify genes upregulated or downregulated in mutant genotypes (Fig. 5*G*). Expression of genes involved in inflammatory responses was not enriched in *Atm*^*Δ/Δ*^ HSCs (data not shown), although physiologically aged HSCs (2 years old) showed higher expression of genes related to inflammatory responses (Fig. 3, *D*–*F*). We confirmed that *p38α* suppresses transcriptional signatures associated with the inflammatory responses and TNFα responses independent of *Atm* deficiency (Fig. 5, H and I). Also, *p38α* deficiency was associated with loss of HSC markers and gain of lineage markers relative to both WT and *Atm*^*Δ/Δ*^ HSCs, suggesting that *p38α* maintains stemness independently of *Atm* deficiency (Fig. 5, *J* and *K*). In addition, loss of *p38α* upregulated genes involved in the PI3K/Akt/mechanistic target of rapamycin pathway (compared with expression in WT and *Atm*^*Δ/Δ*^ HSCs) (Fig. 5*L*). Taken together, these findings suggest that p38α promotes inflammation and maintains HSC function independently of *Atm* deficiency, and that these transcriptional changes support the results of defects in *Atm*^*Δ/Δ*^*p38α*^*Δ/Δ*^ HSCs (Fig. 4, *F*–*K*).

## Discussion

Organismal aging is a multifaceted process mediated in part by aging of somatic stem cells. In hematopoiesis, HSC aging is characterized by cellular phenotypes, including increased pool size, defective transplantation capacity, skewing of differentiation, and reduced clonality (4, 5, 6, 7, 8). The molecular basis of these changes in aged HSCs has been investigated in recent years. In terms of cell-autonomous mechanisms, accumulation of DNA damage, telomere shortening, mitochondrial dysfunction, impaired autophagy, epigenetic reprogramming, and loss of cellular polarity are implicated in HSC aging (4, 6, 25). Cell-extrinsic factors reported to regulate HSC aging include niche-derived factors (26, 27, 28). Suppression of many of these activities at least partly restores aging phenotypes; however, neither how aged HSCs acquire these properties remain clear nor is it known whether they are acquired gradually or *via* a multistepped process.

Previous studies identified p38MAPK as a candidate signal inducing HSC aging *in vivo* (29), as treatment of HSCs or mice with p38MAPK inhibitors reduces aging-related HSC phenotypes. These analyses suggest that p38MAPK functionally impairs HSCs (13, 22). Here, we assessed p38MAPK function in a mouse model of aging by genetic knockout of p38α at either early or late time points followed by analysis of early or late progression phases of chronological aging. Analysis of 1-year-old mice that had been made deficient in *p38α*^*Δ/Δ*^ at 6 to 10 weeks of age (a model for the early progression phase) showed that HSC repopulation capacity did not improve relative to controls but rather declined (Fig. 1, *D*–*J*), whereas HSCs derived from 2-year-old mice that had been made deficient in *p38α* approximately 1 year earlier (a model for the late progression phase) showed partially improved PB and BM chimerism relative to WT controls (Figs. 2, D–I and S2, *J* and *K*). In terms of differentiation skewing, *p38α* loss in the early progression phase of aging increased granulocytic and CD4^+^ T cell differentiation but reduced B cell differentiation at steady state (Fig. 1*B*). In HSC transplantation models of the early progression phase, primary recipients exhibited normal differentiation status, but secondary recipients showed increased T cell output and decreased myeloid output (Fig. 1*H*). In contrast, differentiation status of HSCs from the late progression model was almost identical at steady state or after transplantation (Figs. 2, *B*, *C*, *E*, and *H* and S2, *H* and *I*). These results suggest overall that p38MAPK is indispensable for HSC repopulation capacity or proper differentiation at early progression phases but is not necessary or may even impair HSC activity in the late progression phase of chronological aging.

Previously, we reported that p38α activates purine metabolism to promote stress-induced proliferation of young HSCs after transplantation or BM recovery. The repopulation ability of 1-year-old *p38α*^*Δ/Δ*^ HSCs was similar to that of young *p38α*^*Δ/Δ*^ HSCs, as we previously reported; however, the repopulation capacity of 2-year-old *p38α*^*Δ/Δ*^ HSCs was better than that of 2-year-old *p38α*^*+/+*^ HSCs. To understand why p38α function differs with age, we performed transcriptome analysis. We found that genes related to inflammation, including interferon and TNFα responses, were significantly upregulated in 2-year-old HSCs compared with middle-aged HSCs but not in middle-aged or young HSCs (Figs. 3, *E* and *F* and S3*E*). In addition, enrichment of inflammatory genes in *p38α*^*Δ/Δ*^ HSCs from 2-year-old mice was significantly lower than that in *p38α*^*+/+*^ HSCs from 2-year-old mice (Figs. 3, *G* and *H* and S3*G*), suggesting that *p38α* makes a greater contribution to the inflammation-prone phenotype during the late phase of aging (from 1 to 2 years old) than in the early phase (from young to 1 year old). Aging-associated inflammatory cytokines such as TNFα and interleukin-1 suppress HSC function (30, 31). We found that gene sets related to TNFα signaling *via* p38 were downregulated in 2-year-old *p38α*^*Δ/Δ*^ HSCs relative to 2-year-old *p38α*^*+/+*^ HSCs (Fig. 3*G*). Indeed, loss of *p38α* from 2-year-old HSCs improved their repopulation capacity following transplantation (Fig. 2, G and I) and upregulated gene signatures related to young HSC (Fig. 3*I*). In contrast to those in young HSCs, these findings suggest that p38α makes a positive contribution to inflammation during the late phase aging, resulting in defects in 2-year-old HSCs. Future studies may clarify the events downstream of p38MAPK during the late phase of aging.

*Atm* loss induces HSC defects, including decreased number of HSCs and transplantation defects resembling phenotypes of premature aging. Previous studies showed that the ROS–p38MAPK–p16^Ink4^/p19^Arf^ pathway induces HSC dysfunction in *Atm*^*Δ/Δ*^ mice (22, 23). Here, we found that *Atm*-deficient mice doubly deficient in *p38α* showed reduced numbers of progenitors at steady state and a decrease in donor-derived PB and BM chimerism after transplantation relative to *Atm* single knockouts (Figs. 4, F–I and S4*B*). We conclude that *p38α* loss does not rescue but rather can worsen hematological phenotypes seen in *Atm*^*Δ/Δ*^ mice. Interestingly, we did not observe activation of ROS–p38MAPK–p16^Ink4^/p19^Arf^ signaling by inducible deletion of *Atm in vivo* (Fig. 5, *A*–*D*). Previous studies have analyzed mice with germ line *Atm* knockout (22, 23); thus embryonic or neonatal *Atm* loss of HSCs may have induced the ROS/p38MAPK/*Ink4a–Arf* pathway in HSCs in that context. Consistent with previous reports (22, 32), we observed here that *Atm*^*Δ/Δ*^ HSCs accumulate DNA damage (Fig. 4, *A*–*C*). Although we used *Atm*^*Δ/Δ*^ mice as a model of premature aging, HSCs from these mice did not show enrichment of inflammatory genes (as opposed to HSCs from physiologically aged mice) (data not shown). Loss of *p38α* downregulated expression of inflammatory gene signatures, even in *Atm*^*Δ/Δ*^ HSCs; however, *p38α* deficiency did not rescue HSC function after serial transplantation (Fig. 4, *G*–*K*) or rescue inflammatory gene signatures in *Atm*^*Δ/Δ*^ HSCs (Fig. 5*J*). These findings suggest that *Atm* deficiency is not identical to physiological aging, although *Atm* deficiency reportedly mimics physiological aging; thus, *p38α* deficiency induces different effects.

Previous reports suggest that p38MAPK mediates acquisition of HSC aging phenotypes induced by hematological stresses, such as chronological aging, ROS, or BMT (13, 23, 29, 33). In some reports, treatment of *Atm*^*Δ/Δ*^ mice or aged WT mice with the p38MAPK inhibitor SB203580 or a dominant negative form of a kinase upstream of p38MAPK (13) abrogated HSC aging phenotypes (13, 23). Different outcomes reported by us and those studies may be attributable to off-target effects of the p38MAPK inhibitor, nonenzymatic function of p38MAPK, or to other p38 isozymes.

In summary, our study reveals multifaceted functions of p38MAPK in various contexts of HSC aging: p38α functioned differently in early *versus* late progression phases of chronological aging and in an *Atm*-deficient model of premature aging. These findings provide novel insight into hematopoietic and systemic aging from the viewpoint of HSC regulation.

## Experimental procedures

### Experimental model and subject details

#### Mice

CAG-CreERT2 mice, *p38α*^*fl/fl*^ mice, and *Atm*^*fl/fl*^ mice (34, 35, 36) were crossed and genotyped using PCR-based assays using tail DNA samples. No specific randomization or blinding protocol was used, and both male and female animals were used indifferently in the study. Cre activation was induced by intraperitoneal injection of 2 mg TAM on 5 consecutive days in *p38*α^*Δ/Δ*^*, Atm*^*Δ/Δ*^, and *Atm*^*Δ/Δ*^*p38α*^*Δ/Δ*^ mice. Age-matched TAM-injected CAG-CreERT2(−)-*p38α*^*fl/fl*^, *Atm*^*fl/fl*^, or *Atm*^*fl/fl*^*p38α*^*fl/fl*^ mice served as controls (p38α^*+/+*^, *Atm*^*+/+*^, or *Atm*^*+/+*^*p38α*^*+/+*^ mice). C57BL/6-Ly5.1 congenic mice were used for competitive repopulation assays. All animal experiments were approved by the animal experiment committee of National Center for Global Health and Medicine Research Institute and performed in accordance with the guidelines of National Center for Global Health and Medicine Research Institute.

### Reagents and antibodies

The following mAbs were used in this study: rat mAbs against CD16/32 (93; eBioscience), c-Kit (2B8; eBioscience and Tonbo Biosciences), Sca-1 (E13-161.7; BioLegend), CD4 (L3T4; BD Biosciences), CD8a (53-6.72; BD Biosciences), B220 (RA3-6B2; BD Biosciences), TER-119 (TER119; Tonbo Biosciences), Gr-1 (RB6-8C5; BD Biosciences), CD34 (RAM34; eBioscience), Mac-1 (M1/70; BD Biosciences and eBioscience), Flt3 (A2F10.1; BioLegend), CD45.2 (104; BD Biosciences), CD45.1 (A20; BD Biosciences), IL7Rα (SB/119; BioLegend), CD41 (MWReg30; BD Biosciences), CD48 (HM48-1; BioLegend), CD150 (TC15-12F12.2; BioLegend), and CD45 (30-F11; BioLegend and eBioscience and BD Biosciences). A mixture of mAbs against CD4, CD8, B220, TER-119, Mac-1, and Gr-1 was used as a lineage marker (lineage). A mouse anti–Ki67-Alexa Fluor 555 antibody (B56; BD Biosciences) was used to assess the cell cycle, and mouse antiphosphorylated-p38MAPK–phenylephrine (PE) antibody (36/p38; BD Biosciences) and mouse anti-IgG1–PE antibody (BD Biosciences) were used to detect phosphorylated p38MAPK by intracellular flow cytometry. Annexin V–PE (556422; BD Bioscience) was used to analyze apoptosis. For immunocytochemistry, an anti-H2AX (pS139) antibody (N1-431; BD Bioscience), a rabbit anti-53BP1 polyclonal antibody (NB100-304; Novus Biologicals), and Alexa Fluor 555–conjugated anti-rabbit IgG (A-21428; Invitrogen) were used.

### Cell preparation and flow cytometry

HSC fractions were analyzed essentially as described previously (37). HSPCs were phenotypically defined as follows: LT-HSCs, lineage^−^Sca-1^+^c-Kit^+^CD34^−^Flt3^−^ or lineage^−^Sca-1^+^c-Kit^+^CD48^−^CD150^+^; short-term HSCs, lineage^−^Sca-1^+^c-Kit^+^CD34^+^Flt3^−^ or lineage^−^Sca-1^+^c-Kit^+^CD48^−^CD150^−^; MPPs, lineage^−^Sca-1^+^c-Kit^+^CD34^+^Flt3^+^; MPP2 cells, lineage^−^Sca-1^+^c-Kit^+^Flt3^−^CD48^+^CD150^+^; MPP3 cells, lineage^−^Sca-1^+^c-Kit^+^Flt3^−^CD48^−^CD150^−^; MPP4 cells, lineage^−^Sca-1^+^c-Kit^+^Flt3^+^; common lymphoid progenitors, lineage^−^Sca-1^low^c-Kit^low^IL-7R^+^Flt3^+^; megakaryocyte/erythroid progenitors, lineage^−^Sca-1^−^c-Kit^+^CD16/32^−^CD34^−^; and granulocyte/macrophage progenitors, lineage^−^Sca-1^−^c-Kit^+^CD16/32^+^CD34^+^. Cell sorting was performed using a SORP FACS Aria IIu or FACS Aria IIIu instrument (BD Biosciences). Data were analyzed with FlowJo software (TreeStar).

### BMT and PB analysis

HSCs (CD150^+^CD48^−^CD34^−^Flt3^−^LSKs) from C57BL/6-Ly5.2 mice, together with 4 × 10^5^ bone marrow–derived mononuclear cells (BMMNCs) from C57BL/6-Ly5.1 mice, were transplanted into C57BL/6-Ly5.1 congenic mice. Donor cells were transplanted retro-orbitally into recipients that had been lethally irradiated (9.5 Gy using MBR-1520R [Hitachi Power Solutions], 125 kV 10 mA, 0.5 mm Al, 0.2 mm Cu filter). At 1, 2, 3, and 4 months after BMT, PB was collected and analyzed as described (22, 38). Four months after BMT, 1 × 10^6^ BMMNCs from primary (secondary) recipients were intravenously transplanted into lethally irradiated (9.5 Gy) secondary (tertiary) recipients (Ly5.1).

### Cell cycle and phosphoflow analysis

For Ki67-based cell cycle analysis, BMMNCs were surface stained to distinguish HSPC fractions. After fixation and permeabilization using Cytofix/Cytoperm Kit (BD Bioscience), according to the manufacturer's protocol, cells were stained with an anti-Ki67 antibody (BD Bioscience) and Hoechst 33342 (Invitrogen), and analyzed by flow cytometry. Analysis of phospholylated-p38MAPK was performed as described (16). In brief, cells were surface stained, fixed, permeabilized, and stained with antiphospho-p38MAPK–PE (BD Biosciences, 612565) antibody. Mouse anti-IgG1–PE antibody (BD Biosciences) served as control. Cells were analyzed by flow cytometry. Phospho-p38MAPK–PE intensity was normalized to the mean fluorescence intensity of the IgG control.

### Mitochondrial ROS and apoptosis analysis

Mitochondrial ROS analysis was performed as described (39). In brief, BMMNCs were surface stained to identify HSPC fractions, prewarmed for 10 min at 37 °C, and then incubated 30 min at 37 °C with MitoTracker Orange CM-H_2_TMROS (Life Technologies; M7511). Cells were then analyzed by flow cytometry. To detect apoptotic cells, BMMNCs were stained using an Annexin V-FITC Apoptosis Detection Kit (BD Biosciences; 51-66121E) according to the manufacturer's instructions and analyzed by flow cytometry.

### Colony-forming assays

On day 0, sorted lineage^−^Sca-1^+^c-Kit^+^ cell (LSK) cells were plated in semisolid methylcellulose medium (MethoCult GF M3434; Stem Cell Technologies) and then incubated at 37 °C in 5% CO_2_. Colonies derived from HSCs were counted on days 7 and 14.

### Complementary DNA synthesis and RT-qPCR

Total RNA was extracted from sorted HSCs (CD150^+^CD48^−^CD34^−^Flt3^−^LSK) using an RNeasy Mini Kit (QIAGEN). Complementary DNA was reverse transcribed using Superscript VILO (Invitrogen). qPCR was performed using SYBR Premix ExTaq IIa (TaKaRa Bio) according to the manufacturer's instructions. Gene expression levels were detected using the ABI 7500 Fast Real-Time PCR System with TaqMan Gene Expression Assay Mixes (Applied Biosystems), under the following conditions: 95 °C for 10 s followed by 40 cycles of 95 °C for 5 s and 60 °C for 34 s. Expression levels were determined as 2ˆ(Ct value − mean Ct value of β-actin) and normalized against those seen in control samples, unless otherwise stated.

### Immunocytochemistry after BMT

BMMNCs (5 × 10^5^ cells) from *Atm*^*Δ/Δ*^, *Atm*^*Δ/Δ*^*p38α*^*Δ/Δ*^, and *Atm*^*+/+*^*p38α*^*+/+*^ mice were transplanted into lethally irradiated C57BL/6-Ly5.1 congenic mice. One week after BMT, donor-derived HSC fractions were sorted from recipient BMMNCs. Sorted cells were cytospinned, fixed in 4% paraformaldehyde, and blocked with 1% bovine serum albumin (SIGMA; A4503) for 30 min at room temperature. Cells were then incubated with primary antibody overnight at 4 °C, washed in PBS, and incubated with secondary antibody for 1 h at room temperature. Nuclei were identified by 4′,6-diamidino-2-phenylindole staining (Invitrogen). Samples were analyzed using a laser-scanning confocal microscope (FV-1000; Olympus). Primary and secondary antibodies used plus dilutions as follows: Alexa Fluor 488 mouse anti-γH2AX (BD Bioscience; 560445; 1:20), rabbit antimouse 53BP1 (Novus Biologicals; NB100-304; 1:200), and Alexa Fluor 555 goat anti-rabbit IgG (Invitrogen; A21428; 1:200).

### Microarray analysis

HSCs (CD150^+^CD41^−^CD48^−^CD34^−^Flt3^−^LSK) were sorted into S-Clone SF-O3 medium (Iwai; 1303) and then centrifuged at 340*g* for 5 min at 4 °C and lysed with 75 μl RLT buffer (Qiagen) + 0.75 μl 2-mercaptoethanol. RNA extraction, complementary DNA synthesis, microarray analysis, and data normalization were outsourced to DNA Chip Research, Inc. Normalized expression data were analyzed using GSEA, version 2.0.13 software (Broad Institute). Most gene sets were obtained from the Molecular Signatures Database, version 4.0 distributed at the GSEA Web site (http://www.broadinstitute.org/gsea/index.jsp). See Table S4 for details. Gene sets with a nominal *p* value <0.05 and a false discovery rate *q* value <0.25 were considered statistically significant.

### Statistical analysis

Data are presented as the means ± SD, unless stated otherwise. Statistical significance was determined by Tukey's multiple comparison test. A two-tailed Student's *t* test was used to compare two-group experiments (∗*p* < 0.05, ∗∗*p* < 0.01, and ∗∗∗*p* < 0.001, and ns, not significant).

## Data availability

Raw data of microarray analysis are available at National Center for Biotechnology Information-Gene Expression Omnibus under accession number GSE168057 and GSE168085.

## Supporting information

This article contains supporting information.

## Conflict of interest

The authors declare that they have no conflicts of interest with the contents of this article.
