# Supplementary material for: p38α plays differential roles in hematopoietic stem cell activity dependent on aging contexts
Source: J Biol Chem. 2021 Mar 18;296:100563. doi: 10.1016/j.jbc.2021.100563 (PMC8065231; doi:10.1016/j.jbc.2021.100563)
Supplement: Figures S1 to S5 [file mmc5.docx]

# **Supporting Information**

**p38α plays differential roles in hematopoietic stem cell activity dependent on aging contexts**

Yuriko Sorimachi, Daiki Karigane, Yukako Ootomo, Hiroshi Kobayashi, Takayuki Morikawa, Kinya Otsu, Yoshiaki Kubota, Shinichiro Okamoto, Nobuhito Goda, and Keiyo Takubo

**Material included:**

Supplementary Figures and Legends

**
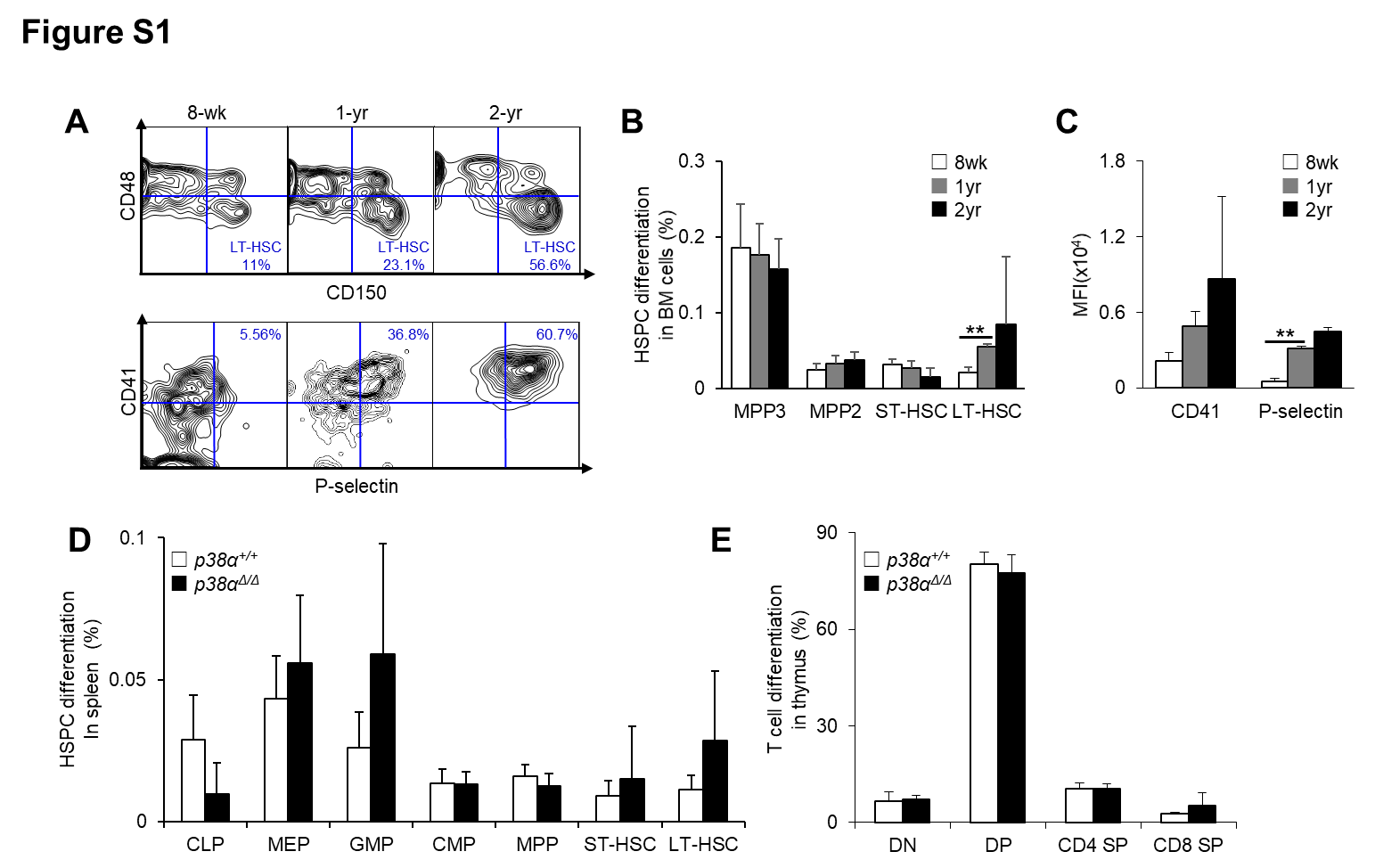
**

**Figure S1, related to Figure 1.**

(**A**) Flow cytometry analysis of hematologic phenotypes in 8-week- (8 wk), 1-year- (1 yr), and 2-year-old (2 yr) wild-type mice. Representative contour plots of LT-HSCs (CD48^-^CD150^+^LSK) (upper) LT-HSCs (CD41 and P-selectin expression) (lower). (**B** and **C**) Percentages of BM HSPCs (LT-HSCs (CD48^-^CD150^+^LSK), ST-HSCs (CD48^-^CD150^-^LSK), MPP2 (CD48^+^CD150^+^LSK), and MPP3 (CD48^+^CD150^-^LSK) (**B**) cells, and expression levels of CD41 and P-selectin in LT-HSCs (**C**) in wild-type mice aged 8 weeks, 1 year, and 2 years. Mean fluorescence intensity (MFI) was analyzed by flow cytometry (means ± SD, n = 2-8). (**D**) Percentage of HSPCs, including CLPs (common lymphoid progenitors; Lin^-^IL7Rα^+^Flt3^+^Sca-1/c-Kit^lo^), MEPs (megakaryocyte-erythroid progenitors; Lin^-^IL7Rα^-^Sca-1^-^c-Kit^+^CD16/32^-^CD34^-^), GMPs (granulocyte-monocyte progenitors; Lin^-^IL7Rα^-^Sca-1^-^c-Kit^+^CD16/32^+^CD34^+^), CMPs (common myeloid progenitors; Lin^-^IL7Rα^-^Sca-1^-^c-Kit^+^CD16/32^-^CD34^+^), MPPs (CD34^+^Flt3^+^LSK), ST-HSCs (CD34^+^Flt3^-^LSK), and LT-HSCs (CD34^-^Flt3^-^LSK), in the spleen of *p38α^+/+^* or *p38α^Δ/Δ^* mice aged 1 year (mean ± SD, n = 5) .

(**E**) Percentage of thymic T cells, including double negative (DN; CD4^-^CD8^-^), double positive (DP; CD4^+^CD8^+^), CD4^+^CD8^-^ (CD4 SP), and CD4^-^CD8^+^ (CD8 SP) cells, in *p38α^+/+^* or *p38α^Δ/Δ^* mice aged 1 year (means ± SD, n = 5). All data are derived from a single experiment. **P < 0.01.

**
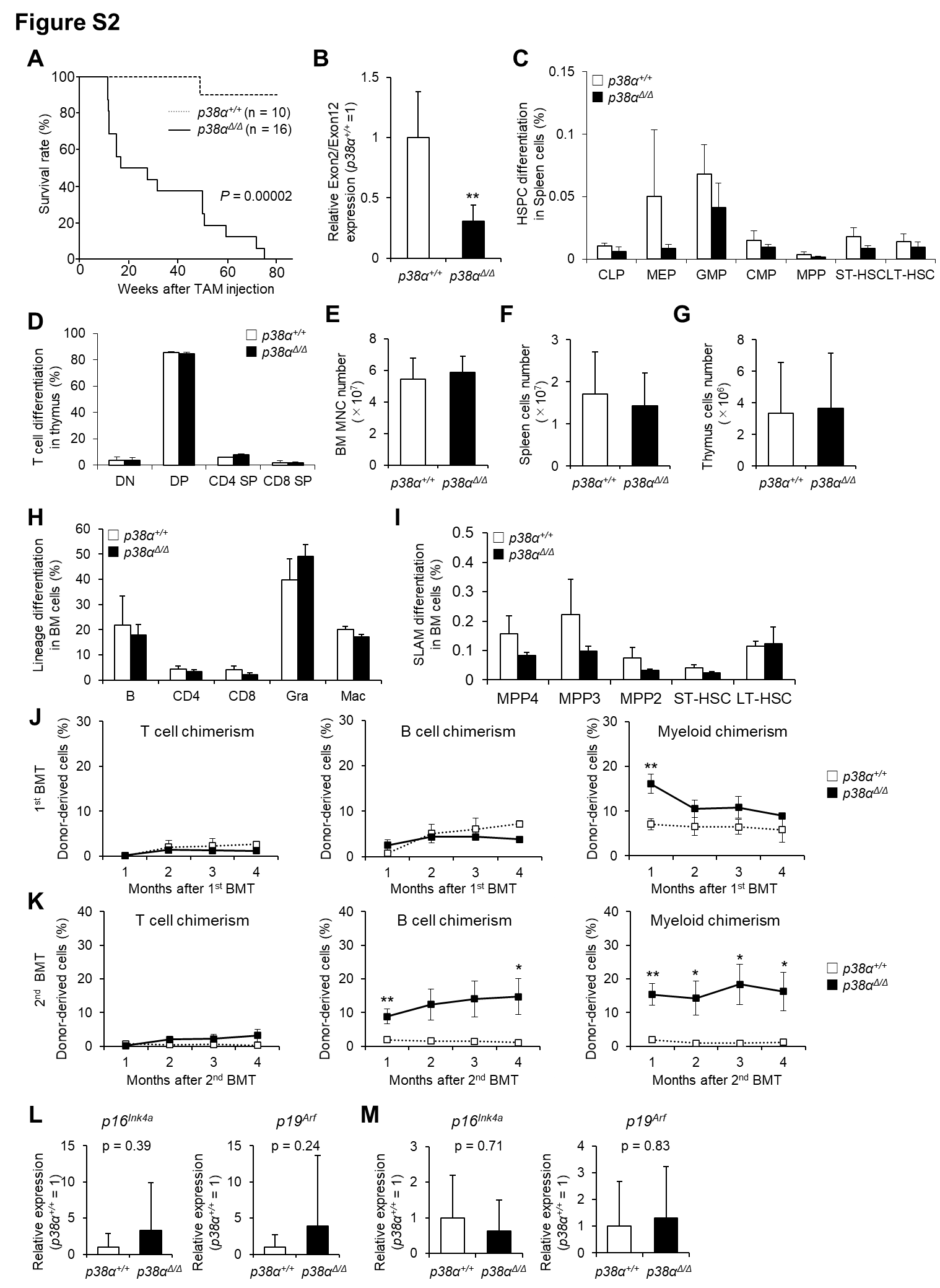
**

**Figure S2, related to Figure 2.**

(**A**) Survival of *p38α^+/+^* or *p38α^Δ/Δ^* mice following tamoxifen (TAM) injection (n = 10-16). TAM was injected into mice aged 10 to 25 weeks. (**B**) Quantification of *Mapk14* deletion efficiency by genomic qPCR of *p38α^+/+^* or *p38α^Δ/Δ^* PB cells from 2-year-old mice injected with TAM injection approximately 1 year earlier (mean ± SD, n = 2-9). (**C**) Percentage of HSPCs, including CLPs (common lymphoid progenitors; Lin^-^IL7Rα^+^Flt3^+^Sca-1/c-Kit^lo^), MEPs (megakaryocyte-erythroid progenitors; Lin^-^IL7Rα^-^Sca-1^-^c-Kit^+^CD16/32^-^CD34^-^), GMPs (granulocyte-monocyte progenitors; Lin^-^IL7Rα^-^Sca-1^-^c-Kit^+^CD16/32^+^CD34^+^), CMPs (common myeloid progenitors; Lin^-^IL7Rα^-^Sca-1^-^c-Kit^+^CD16/32^-^CD34^+^), MPPs (CD34^+^Flt3^+^LSK), ST-HSCs (CD34^+^Flt3^-^LSK), and LT-HSCs (CD34^-^Flt3^-^LSK), in the spleen of 2-year-old *p38α^+/+^* or *p38α^Δ/Δ^* mice (mean ± SD, n = 3-5).

(**D**) Percentage of thymic T cells, including double negative (DN; CD4^-^CD8^-^), double positive (DP; CD4^+^CD8^+^), CD4^+^CD8^-^ (CD4 SP), and CD4^-^CD8^+^ (CD8 SP) in 2-year-old *p38α^+/+^* or *p38α^Δ/Δ^* mice (mean ± SD, n = 2–3). (**E-G**) Number of MNCs in the BM (**E**), spleen (**F**), and thymus (**G**) in 2-year-old *p38α^+/+^* or *p38α^Δ/Δ^* mice (mean ± SD, n = 2-3).

(**H** and **I**) Analysis of BM differentiation status (B220^+^ B cells [B], CD4^+^ T cells [CD4], CD8^+^ T cells [CD8], Mac-1^+^Gr-1^hi^ granulocytes [Gra], and Mac-1^+^Gr-1^lo^ macrophages [Mac]) (mean ± SD, n = 7-9) (**H**), and percentage of BM SLAM, including MPP4 (Flt3^+^LSK), MPP3 (Flt3^-^CD150^-^CD48^+^LSK), MPP2 (Flt3^-^CD150^+^CD48^+^LSK), ST-HSCs (Flt3^-^CD150^-^CD48^-^LSK), and LT-HSCs (Flt3^-^CD150^+^CD48^-^LSK) (mean ± SD, n = 3-5) (**I**), in 2-year-old *p38α^+/+^* or *p38α^Δ/Δ^* mice. (**J** and **K**) Chimerism of donor-derived differentiated cells (CD4/8+ T cells [T], B220+ B cells [B], and Gr-1/Mac-1+ myeloid cells [Myeloid]) in PB cells from primary (**J**) and secondary (**K**) recipients (mean ± SE, n = 7-8). (**L** and **M**) Expression of *p16^Ink4a^* and *p19^Arf^* in *p38α^+/+^* or *p38α^Δ/Δ^* steady state LT-HSCs (CD34-Flt3- LSK) (**L**) and secondary transplanted LT-HSCs (CD34-Flt3-LSK) (**M**), as assessed by qPCR (mean ± SD, n = 4-19).

All data are derived from a single experiment. *P < 0.05, **P < 0.01.


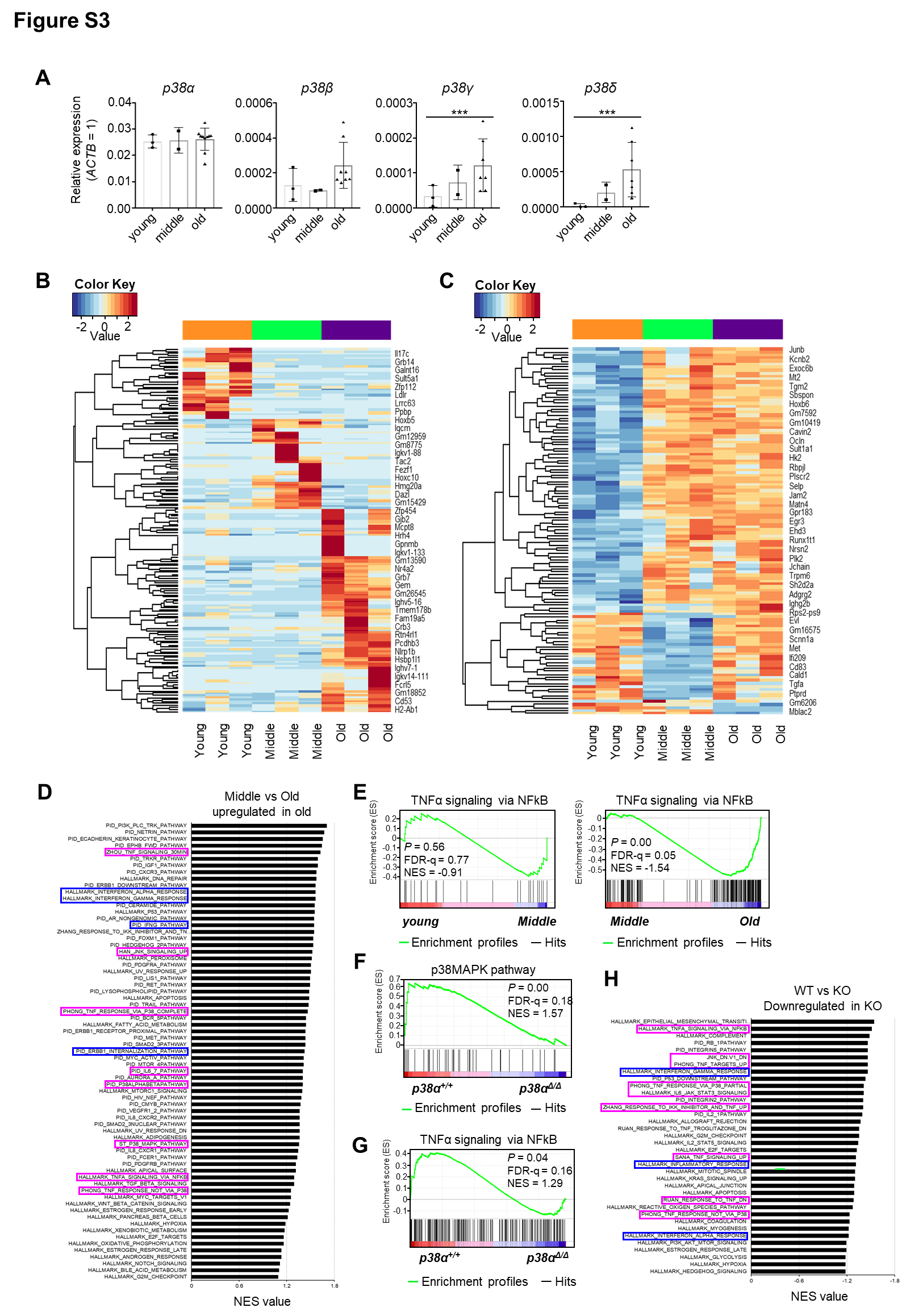


**Figure S3, related to Figure 3**

(**A**) RT-PCR analysis of p38 isozymes other than p38α (i.e., p38β, p38γ, and p38δ) in young (10-week), middle-aged (1-year) and old (2-years) LT-HSCs (CD48^-^CD150^+^LSK). Data are expressed as -fold induction relative to expression of β-actin (*ACTB*) (mean ± SD, n = 2-10). (**B** and **C**) Heatmap of the genes specifically up- (**B**) or downregulated (**C**) in HSCs from 2-month-old (Young, light green) or 12-month-old (Middle, light green) or 24-month-old (Old, purple) mice. The heatmap shows the z-normalized score of each sample; genes were selected on the basis of a BH p-value < 0.0015 or a FDR q-value < 0.25. The heatmap shows representative genes; all genes are shown in Tables S1 and S2. The color key indicates an increase (or decrease) in gene expression in HSCs according to age. (**D**) Gene sets enriched in 2-year-old compared with 1-year-old LT-HSCs (CD48^-^CD150^+^LSK). Bar graph shows the negative Normalized Enrichment Score (NES).

(**E**) GSEA plots of young (2 months), middle-aged (12 months) and old (24 months) HSCs to identify genes involved in TNFα responses via NF-κB. (**F**) GSEA analysis of *p38α^+/+^* or *p38α^Δ/Δ^* LT-HSCs (CD48^-^CD150^+^LSK) for genes related to the p38MAPK pathway. (**G**) GSEA plots of *p38α^+/+^* or *p38α^Δ/Δ^* LT-HSCs from 2-year-old mice to identify genes involved in TNFα responses via NF-κB.

(**H**) Gene sets downregulated in *p38α^Δ/Δ^* compared with *p38α^+/+^* LT-HSCs (CD48^-^CD150^+^LSK). The bar graph shows the negative Normalized Enrichment Score (NES). All data are derived from a single experiment. ***P < 0.001.


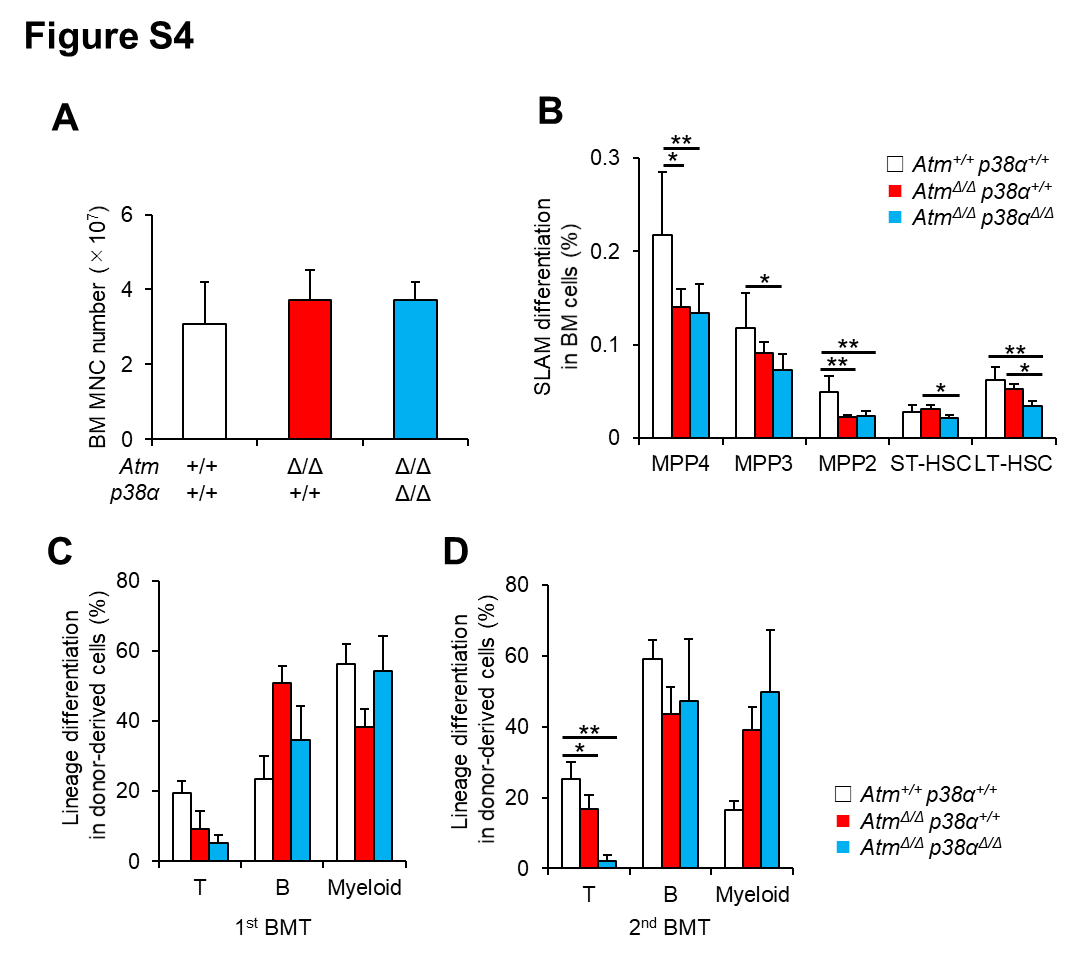


**Figure S4, related to Figure 4.**

(**A**) MNC number in the BM (means ± SD, n =5). (**B**) Percentage of MPP4 (Flt3^+^LSK), MPP3 (Flt3^-^CD150^-^CD48^+^LSK), MPP2 (Flt3^-^CD150^+^CD48^+^LSK), ST-HSCs (Flt3^-^CD150^-^CD48^-^LSK), and LT-HSCs (Flt3^-^CD150^+^CD48^-^LSK) in the BM of mice of the indicated genotypes (mean ± SD, n = 5). (**C** and **D**) Lineage differentiation (CD4/8^+^ T cells [T], B220^+^ B cells [B] or Gr-1/Mac-1^+^ myeloid cells [Myeloid]) in PB cells from primary (**C**) and secondary (**D**) recipients at 4 months post-BMT (mean ± SE, n = 4-6). All data are derived from a single experiment. *P < 0.05, **P < 0.01.


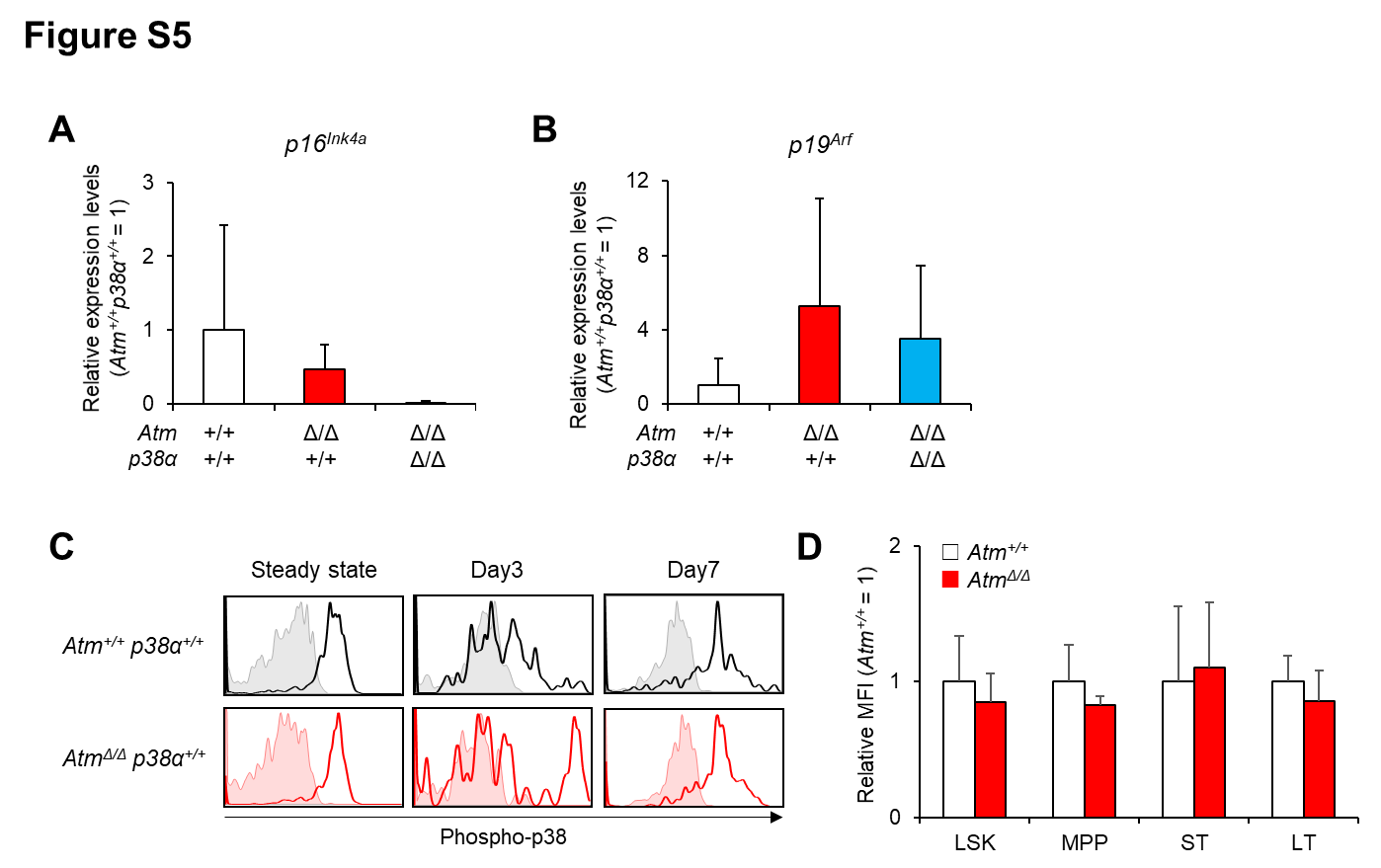


**Figure S5, related to Figure 5.**

(**A** and **B**) Expression of *p16^Ink4a^* (**A**) and *p19^Arf^* (**B**) in LT-HSCs (CD34^-^Flt3^-^LSK) of the indicated genotypes (mean ± SD, n = 4). (**C**) Representative histograms showing data showing p38MAPK phosphorylation status in LT-HSCs (CD34^-^Flt3^-^LSK) from mice of the indicated genotypes at steady state, and at 3 and 7 days after receiving 4 Gy radiation. (**D**) p38MAPK phosphorylation status in LT-HSCs (CD34^-^Flt3^-^LSK), ST-HSCs (CD34^+^Flt3^-^LSK), MPPs (CD34^+^Flt3^+^LSK), LSK (Lin^-^c-Kit^+^Sca-1^+^) from mice of the indicated genotypes at 7 days post-transplantation. Mean fluorescence intensity (MFI) was analyzed by intracellular flow cytometry (means ± SD, n = 4). All data are derived from a single experiment.
